# Scaling Up Access to Implants: A Summative Evaluation of the Implants Access Program

**DOI:** 10.9745/GHSP-D-19-00383

**Published:** 2020-06-30

**Authors:** Rebecca Braun, Annika Grever

**Affiliations:** a Global Impact Advisors, San Mateo, CA, USA.

## Abstract

The Implants Access Program (IAP) was a partnership between public and private organizations that aimed to increase access to contraceptive implants for women in low-income countries. The partnership began with 2 volume guarantee agreements that reduced the price of implants by approximately 50% and was complemented by efforts to address supply chain, service delivery, and knowledge and awareness barriers. We conducted a summative evaluation to identify key insights related to the IAP’s relevance, effectiveness, and sustainability. We completed a desk review of program materials and published literature, followed by 42 in-depth interviews, including global stakeholders and country stakeholders in 3 case example countries: Kenya, Nigeria, and Uganda. The evaluation found evidence of increased access to implants including a 10-fold increase in procurement between 2010 and 2018 and an increase in prevalence of contraceptive implants during this same period. The IAP leveraged global family planning efforts taking place at the time, and its partnerships offered a business case for manufacturers to support increased access to implants. Enhanced supply chain visibility and coordination helped limit country-level stock-outs, and the IAP built on existing in-country delivery capacity. Although the IAP was able to address key challenges due to its effective collaboration and coordination at global and country levels, sustaining progress requires institutionalized mechanisms to continue global efforts and long-term assurances that the low price of implants will be maintained. Over 6 years, the IAP supported tremendous progress in increasing access to implants for women in low-income countries by building a public- and private-sector collaboration that focused on systems change in the family planning field. This partnership matched a unique response to a unique problem: building tools, systems, and capacity that can inform and support the introduction and scale-up of new and underutilized contraceptive methods.

## Abstract (precis)

The Implants Access Program increased access to implants by addressing price, supply chain, service delivery, and knowledge and awareness barriers. Sustaining progress requires institutionalized mechanisms to continue global efforts and long-term assurances that implants’ low price will be maintained.

## Teaser

- Devising an innovative solution to address price barriers
- Enhancing supply chain data visibility and coordination to limit stock-outs
- Leveraging existing delivery capacity and strengthening capacity in targeted areas
- Coordinating global and country-level stakeholders to address key challenges

**Key Implications:**

- Donors, policy makers, and implementing partners should align method-specific efforts to support broader family planning goals and implementation plans at the global and country level.
- Donors should engage private-sector manufacturers to identify mutually beneficial opportunities for collaboration to address price barriers.
- National stakeholders and implementing partners should prioritize efforts to improve supply chain visibility, increase efficiency and sustainability of provider training, and support community sensitization and awareness raising to ensure clients can exercise free and informed contraceptive method choice.

## BACKGROUND

At the London Summit on Family Planning in 2012, leaders across the globe committed to providing access to modern contraception to 120 million additional women who want to prevent or delay pregnancy in 69 of the world’s poorest countries by 2020.^1^ Increasing access to long-acting reversible contraceptives (LARCs), which include implants and intrauterine devices (IUDs), presented a critical opportunity to support global efforts to reach this goal.^2^ In the few years before 2012, overall demand for implants began to increase significantly in developing countries,^3^ yet barriers to access remained, including high cost of commodity, few trained providers, and limited supply.^4^^–^^6^

To make contraceptive implants more available to women in the world’s poorest countries, a group of public and private organizations, including the Bill & Melinda Gates Foundation; the Clinton Health Access Initiative (CHAI); the governments of Norway, Sweden, the United Kingdom, and the United States; and the Children’s Investment Fund Foundation, with support from the United Nations Population Fund (UNFPA), formed the Implants Access Program (IAP) in 2013.^7^ The IAP partners sought to increase access to implants by addressing key barriers at the global and country level (Table 1). Specifically, the IAP supported a multipronged effort starting with volume guarantee (VG) agreements with 2 pharmaceutical manufacturers that reduced the price of commodities by approximately 50%. The 2 VGs were backed by the Bill & Melinda Gates Foundation, the governments of Norway and Sweden, and the Children’s Investment Fund Foundation, who agreed to annual minimum purchase volumes that would be met at the reduced price.^8^ These funders partnered with a broader donor group including the funders who procure the majority of contraceptive commodities for FP2020 countries. The reduced commodity price was available to entities serving the poorest women, including governments in FP2020 countries, donors who procured for public-sector or social marketing organization (SMO) delivery in these countries, and some nongovernmental organization/SMO programs. The price agreements were complemented by efforts to address supply chain, service delivery, and knowledge and awareness barriers.^6^

**TABLE 1.** Implants Access Program Objectives, Barriers Addressed, and Partner Approaches

**Objectives**	**Barriers Addressed**	**Partner Approaches**
**1. Improve market dynamics**	High unit price of the primary LARC demanded in FP2020 countries	Volume guarantee to lower price of implants Support for market entry of a generic implant product
**2. Strengthen supply chain performance**	Limited and inconsistent information on country procurement needs and supply availability Inconsistent supply availability at service delivery points	Improvements to data visibility, transparency, and coordination to better match country-level supply and demand Introduction of dashboards and job aids to strengthen and support in-country supply chain efforts
**3. Improve and expand service delivery**	Shortage of trained providers to insert and remove implants	Creation and expansion of innovative and cost-effective training approaches Expansion of the range of service delivery models to provide LARCs
**4. Increase knowledge and awareness**	Limited knowledge among women about family planning options including implants	Community awareness and sensitization activities to increase understanding of family planning and benefits of LARCs
5. Together, the strategies above contributed to a fifth objective: **Improve the enabling environment for contraceptives**		

Abbreviation: LARC, long-acting reversible contraceptive.

**FIGURE 1.**
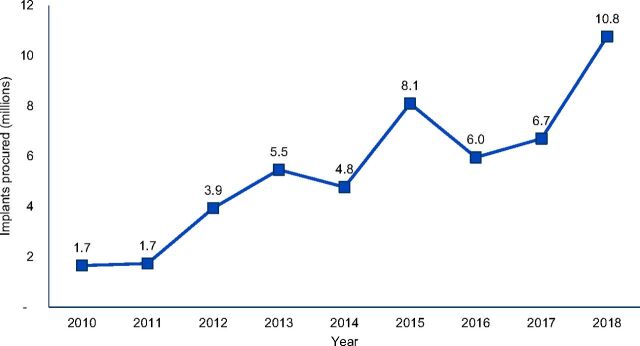
Implant Procurement for FP2020 Countries, 2010–2018^a^ ^a^ These data were sourced from United Nations Population Fund Reproductive Health Interchange on July 1, 2019. Data are provided by the central procurement offices of large family planning donors, institutional buyers, and other organizations that procure contraceptives. The data reflect ∼80% of donor-provided contraceptive supplies and do not include directly procured products by governments. More information is available at: https://www.unfpaprocurement.org/rhi-home.

Overall, the IAP successfully contributed to increased access to implants among women in supported countries. Procurement and use of implants were used as proxies for access for the purpose of this evaluation. Annual procurement of implants for the world’s 69 poorest countries (i.e., FP2020 focus countries) increased 10-fold, from 1.7 million units in 2010 to 10.8 million units in 2018 (Figure 1),^9^ without evidence of overstocking.^10^ The agreed-upon price reductions enabled more than $500 million in cost savings when compared to the cost of procurement at the previous price.^11^ Moreover, contraceptive implant prevalence dramatically increased during that time. A recent analysis of contraceptive implant use across 12 sub-Saharan African countries demonstrated that prevalence rates increased from an average of 1.9% across surveys between 2008 and 2013 to an average of 8.1% across surveys between 2015 and 2017.^5^ Further, in 11 of the 12 sub-Saharan countries with data available from multiyear national surveys, implants use was the primary factor contributing to increases in modern contraceptive prevalence rates between 2003 and 2017, providing a greater contribution than all other modern methods (e.g., pills, injectables and intrauterine devices) combined.^5^

**FIGURE 2.**
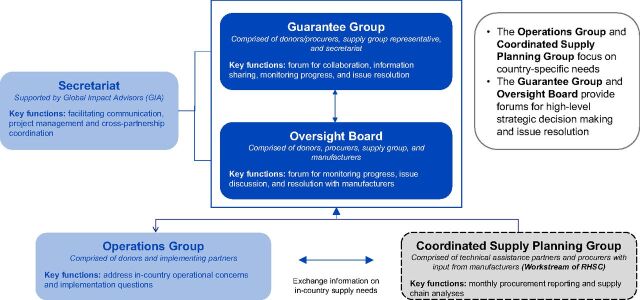
Implants Access Program Governance Structure

The IAP established a governance structure to coordinate activities and enable information sharing (Figure 2). This included the guarantee group, a forum for high-level decision making as well as issue discussion and resolution, and 2 oversight boards that engaged each of the 2 World Health Organization (WHO) prequalified implant manufacturers. The partnership evolved to include a secretariat and was supported by 2 additional groups that focused on country-level needs: the Operations Group and the coordinated supply planning (CSP) group, that had been initiated prior to the IAP in 2012 as a workstream of the Reproductive Health Supplies Coalition (RHSC).

The CSP group aims to prevent family planning commodity stock imbalances by using shared supply chain data and information to coordinate shipments and the allocation of commodities within and among countries. At the country level, IAP partners provided targeted funding and technical support for scale-up efforts through implementing organizations working with public and private sectors, including CHAI, EngenderHealth, Jhpiego, and John Snow, Inc. (JSI), as well as through SMOs, including Marie Stopes International and Population Services International.

The Operations Group was formed in 2015 in response to the need for coordination and support to countries with the transition from Implanon Classic to Implanon NXT. This group supported coordination and communication around global and country-level investments in training and service delivery.

### Evaluation Objectives

The objective of this evaluation was to understand the IAP partnership’s contribution toward achieving increased access to implants, including successes and challenges that were faced, and to identify lessons from the program that could inform future efforts to introduce and scale new and underutilized contraceptive products. We selected an evaluation framework based on the Organisation for Economic Co-operation and Development’s Development Assistance Committee criteria, focusing on relevance, effectiveness, and sustainability.

Accordingly, this study aimed to evaluate the extent to which the IAP responded to the needs of the family planning community, understand progress toward IAP’s original objectives and other unexpected outcomes, and identify the critical factors as well as risks to sustaining its achievements at both global and country levels.

This study evaluated the IAP’s relevance and effectiveness and identified key risks to sustaining progress.

## METHODS

We conducted a summative evaluation from January to June 2019. We started with a desk review of background materials and other relevant documents to provide a deeper understanding of the IAP and guide the key informant interviews. Documents included internal reports and formal meeting summaries produced by IAP partners, published peer-reviewed and gray literature, and notes from interviews with IAP partners conducted in 2017 as part of a sustainability assessment.

After the desk review, we conducted 3 rounds of semistructured in-depth qualitative interviews with 42 stakeholders to answer our questions at global and country levels and to identify specific in-country examples. The first round of 12 interviews included donors, manufacturers, procurers, and technical assistance providers who could provide a global perspective on the IAP. The second round of 10 interviews included implementing partners who could provide a multicountry perspective. The third round of 20 interviews included Ministry of Health (MOH) representatives, procurers, and implementing partners in 3 case-example countries—Kenya, Nigeria, and Uganda—that were cited most frequently during the second round of interviews. Participants in round 1 were selected purposively, and rounds 2 and 3 were selected via snowball sampling from previous participants. Before interviews began, participants were informed that their identity would remain anonymous, and specific quotes would be anonymized.

Interviews were conducted via phone and administered by the evaluation team. Separate semistructured interview guides were developed for each round of stakeholder interviews. Detailed notes were taken by the evaluation team during each interview; data were analyzed with an iterative, thematic approach. The key insights and recommendations are a synthesis of the desk review and interview findings.

## RESULTS

**FIGURE 3.**
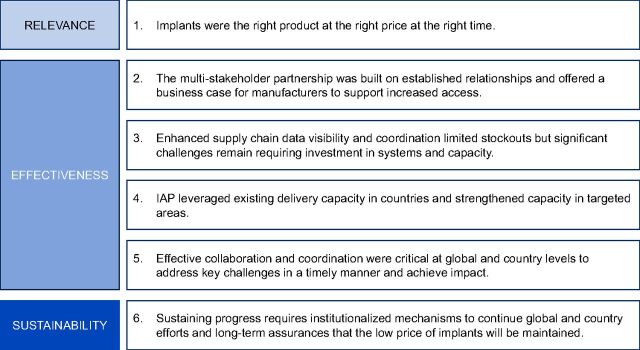
Key Insights from the Implants Access Program Evaluation

The findings that emerged from the IAP evaluation identify factors that contributed to success, challenges that were faced and overcome, as well as challenges that continue to limit progress toward improving access to implants. The findings are framed as 6 key insights related to relevance, effectiveness, and sustainability that can inform future efforts to introduce and scale new and underutilized contraceptive products (Figure 3).

### Relevance

#### 1. Increased Access to Implants

The IAP leveraged global family planning attention and efforts and recognized an opportunity for LARCs and implants specifically. This broader framing and positioning represented a critical evolution of the partnership in response to concerns from the family planning community that the efforts were focused on promoting a single method category.

The timing of the IAP’s launch aligned well with global efforts initiated in 2012, including the London Family Planning Summit, the formation of FP2020, and the United Nations Commission on Life-Saving Commodities for Women and Children report. These efforts aimed to dramatically increase global contraceptive access and options with accompanying new commitments from donors and governments. The IAP leveraged this global momentum and aligned with both the broader family planning ecosystem and emerging global family planning architecture in a way that catalyzed action, as stakeholders could connect the purpose of the program to the global family planning dialogue.

Interviewees noted that the many efforts and events in the family planning field at this time made it difficult to map clear causal linkages to attribute success. However, alignment with the broader family planning ecosystem was seen as critical to success for the IAP. Throughout the IAP’s lifespan, partners were able to draw on the momentum of the global family planning field, building on key events that expanded country and donor commitments to family planning (Table 2).

**TABLE 2.** Key Events in the Global Family Planning Field, 2012–2018

**Event**	**Year**	**Objective**	**Relevance to Implants Access Program**
London Family Planning Summit	2012	This summit secured US$2.3 billion toward meeting the unmet need for contraception for 120 million women worldwide by 2020.	Countries made specific goals around raising modern contraceptive prevalence rate and reducing unmet need; donors committed funding for family planning commodities and service delivery, including implants.
FP2020 launch	2012	This global partnership of governments, donors, civil society organizations, and technical experts emerged to help meet the goals of the 2012 London summit.	FP2020 connected countries committed to LARCs with financial and technical resources as needed.
UN Commodities Commission report	2012	This report listed 13 lifesaving commodities that could save over 6 million lives and avert maternal deaths via improved access to family planning.	Implants were named as a lifesaving commodity and this report identified recommendations to improve financing, utilization, supply, and demand for implants.
UNICEF RMNCH Trust Fund	2013	This fund was established by UNICEF, UNFPA, and WHO to finance high-impact interventions in RMNCH based on recommendations of the UN Commodities Commission report.	The RMNCH trust fund supported eight countries as they expanded the availability of implants and other lifesaving commodities.
WHO task shifting recommendations	2013	The WHO published updated, evidence-based recommendations on the provision of RMNCH interventions by different cadres of health workers.	The updated task shifting recommendations specified that auxiliary nurses and auxiliary nurse midwives should be permitted to insert and remove implants with targeted monitoring and evaluation.
WHO expansion of implants eligibility criteria	2015	The fifth edition of WHO’s Medical Eligibility Criteria reduced restrictions around the use of implants and other hormonal contraceptives for adolescents and breastfeeding women less than 6 weeks’ postpartum.	Postpartum women had more options for hormonal contraceptives, which enabled the opportunity to provide LARCs to women shortly after birth. Adolescents were cleared to access implants.
Youth statement on LARCs	2015	This statement provided evidence that LARCs were safe for youth and adolescents and was signed by over 50 endorsing organizations.	The document provided guidance for programs and service providers that all adolescents and youth deserved access to a full range of methods, including implants.
2017 Family Planning Summit	2017	Donors, policymakers, and advocates convened to assess efforts toward reaching FP2020 goals and accelerate progress.	Countries, donors, civil society organizations, and private sector partners recommitted to LARCs. More than 2 dozen FP2020 countries committed to expanding their method mix.

Abbreviations: LARC, long-acting reversible contraceptive; RMNCH, reproductive, maternal, newborn, and child health; UN, United Nations; UNICEF, United Nations Children’s Fund; UNFPA, United Nations Population Fund; WHO, World Health Organization.

Alignment with the broader family planning ecosystem was seen as critical to IAP’s success.

The IAP also filled a key need identified by global partners to make LARCs, and specifically implants, more available as part of a broader array of contraceptive methods made routinely available to clients in family planning programs. A 2012 United Nations Commission on Life-Saving Commodities for Women and Children report identified implants as one of 3 underutilized family planning products and identified the high price as the key barrier to enable greater access to implants.^4^ One interviewee noted that the link between the IAP and the commission’s recommendations was critical to making a case to donors to support the effort. Implants had also been identified as an underutilized commodity needing attention by the Caucus on New and Underused Reproductive Health Technologies as early as 2011, further confirming the need for attention to this method category.^12^

As a result, IAP partners came together around an innovative solution to the price barrier through 2 VGs that would kickstart the implants market for FP2020 countries.^6^ At the country level, the global momentum and accompanying donor and country commitments to family planning goals were key factors that contributed to the success of the IAP. By the end of 2013, 28 countries had made commitments in support of FP2020. By 2018, 11 countries had identified FP2020 commitments that specifically referenced access to implants as part of expanding contraceptive method choice.^13^ Country-level interviewees noted that political will toward family planning was critical to getting government stakeholders and other partners on board and aligned around a common plan to scale up access to contraceptive methods, including implants.

Ensuring that the IAP strategy aligned with the broader family planning agenda to increase access to a full contraceptive method mix with informed choice and quality service delivery was a key point of alignment in the early phase of forming the partnership. Although this was the approach from the outset at the country level, the global-level framing and positioning of this initiative within the broader family planning agenda was something that evolved during the initial years of the IAP and helped ensure the effort was not perceived as a push from donors for a single contraceptive method.

Although all stakeholders agreed that a price reduction was necessary to unlock demand for implants, there were differing perspectives on whether the agreed price was the right price for a long-term sustainable market. As a manufacturer representative explained, the significant reduction in price and resulting low margin made it difficult to garner their company’s support for investment in manufacturing capacity and limited what they could provide in terms of support to countries for policy change, provider training, and consumer education. The manufacturer noted that this could have an impact on the long-term sustainability of producing the product.

### Effectiveness

#### 2. Addressed Price Barriers

The agreement to reduce prices, which was crucial to the success of the IAP, was made possible through the development of strong personal relationships as well as underlying business fundamentals. The IAP partnership sought to achieve a stable market for implants with sustained affordable prices through 2 VGs, building upon previous price reduction efforts initiated by the RHSC in 2010.^14^ The agreements made Bayer’s Jadelle and Merck’s Implanon (and later Implanon NXT) available to women in the world’s poorest countries at price reductions of approximately 50% through 2018. The aim of the VGs was to increase confidence in long-term demand, allowing manufacturers to make up-front investments that would lower costs and enable reduced prices for years to come.

The IAP also supported ongoing efforts to introduce a prequalified generic product, Dahua’s Levoplant, in collaboration with DKT, to the market to increase competition and maintain the reduced price following the end of the price agreement. Prequalification of the generic product was achieved in 2017 after nearly a decade of effort. IAP partners funded technical assistance to the generic manufacturer and supported market entry through coordinated efforts with countries to consider procurement of the product. All 3 case example countries are planning to introduce Levoplant as an alternative implant. However, several interview participants identified additional provider training requirements as a barrier.

The reduced price achieved through the VGs was critical to scale up access to implants, allowing existing procurement resources to stretch further and dramatically increasing the number of implants procured for FP2020 countries between 2010 and 2018 (Figure 1). Manufacturers have committed to maintaining the new price through 2023, after the VGs formally ended in 2018. Whether the market has reached an equilibrium point with sustainable pricing and supply is yet to be determined and will require continued monitoring over the coming years as the market stabilizes.

The IAP’s engagement with manufacturers was built from established relationships between procurers, donors, and manufacturers who had been working together for many years on a range of family planning products. The VG was presented to manufacturers as a business case with a clear value proposition in terms of increased visibility and stability of long-term demand that would allow suppliers to produce higher volumes at a lower price, while still covering their cost of goods. The agreement was described by a manufacturer representative as a “win-win for all partners engaged with the IAP.” The win for manufacturers was the assurance of sufficient demand (either from procurers or guarantors) to ramp up production and expand capacity while maintaining or improving utilization rates. Further, it was also viewed as a win for manufacturers in terms of their contribution as part of corporate social responsibility and a source of pride for employees working on the product. The win for procurers came from the lower price as well as coordination of orders that emerged from the partnership, allowing them to optimize allocation of available supply and maximize impact. The IAP was also described by manufacturers as a win for women who would now have access to a modern contraceptive method that previously had limited availability and access.

The IAP engaged with manufacturers by building on relationships between procurers, donors, and manufacturers who had worked together for many years on a range of family planning products.

One aspect of the reduced price agreement was to remove most supports for provider training that manufacturers had previously provided, such as training of master trainers or provision of commodities for training (e.g., model arms or placebo implants), thus transferring this financial responsibility to donors and governments. Manufacturers developed and shared global training materials which could be adapted to specific country contexts but, in general, did not provide additional supports. The exception was a limited amount of support that was made available during the transition from Merck’s Implanon Classic to Implanon NXT. Global stakeholders identified the shift in policy regarding training resources as a challenge. There was limited visibility among all partners into the resources that would be required to scale up training for providers as implants procurement increased and how those costs would be covered. The transition to Implanon NXT compounded this problem with additional training requirements. Providers who had already been trained in Implanon Classic required additional training for the updated insertion technology. This training had to be completed during a relatively condensed period as the Implanon Classic was being phased out and replaced by NXT. The intensive resource requirements for this retraining effort fell largely on donors and governments.

#### 3. Enhanced Supply Chain Visibility and Coordination

The IAP built capacity at both the global and country level to address supply chain limitations and limit stock-outs. Although significant challenges remain requiring investment in systems and capacity, the tools developed have been broadened to additional products over time.

To reduce inconsistency of supply availability at the global and country level, the IAP sought to improve both the quality and visibility of the demand pipeline through a mechanism to support procurer and manufacturer coordination and improve associated data. The RHSC’s CSP group addressed this need, mitigating the risk of market disruptions related to the price reduction, such as the potential for overordering and stockpiling of the commodity or identifying where additional funding was needed for procurement to support rapidly growing demand. Through the IAP agreements, manufacturers shared all procurement data with JSI on a monthly basis to monitor progress to goals and identify imbalances between supply and demand. The CSP group contributed to IAP’s effectiveness and brought together key stakeholders who could review shared data on country orders and stocks on hand, identify supply issues that arose, and quickly coordinate action in response. As a manufacturer representative explained:

*CSP group changed interactions. Organizations are now talking to each other, [about] who is delivering what to each country, and now they’re able to go into the field to get a clearer picture, and get more insight into the [demand] forecast.*

The CSP group brought together key stakeholders to review shared data on country orders and stocks, identify supply issues, and coordinate a response.

According to unpublished CSP data from JSI, the CSP coordination in 2018 resulted in recommended actions that avoided national stock-outs and/or shortages of implants in 24 countries, totaling an additional 7.5 million couple years of protection and averting approximately 1.8 million unintended pregnancies.^15^ The effectiveness of CSP was demonstrated early on and the scope quickly expanded to support multiple family planning products.

At the country level, partners developed family planning dashboards to integrate data across service delivery, consumption, and training databases to improve supply availability by targeting available and appropriate commodities at facilities with trained providers, building off of longstanding supply strengthening efforts in the family planning field. The dashboards used in Nigeria and Kenya were created initially in support of implants scale-up efforts, specifically around the transition to NXT. As capacity to use these analytical tools increased, the dashboards were expanded to cover all family planning methods in both countries. The dashboards offered data visibility to stakeholders that previously did not have access to all the data in an integrated manner. Although the dashboards improved implant supply availability and had important benefits for other family planning products as well, they did not solve the supply chain challenges.

Similarly, as part of overall capacity building efforts, implementing partners also developed job aids and training for commodity managers to improve supply planning, and trained health care providers on the importance of data and reporting to improve supply availability. Implementing partners often filled gaps in supply chain systems by facilitating communications between warehouses and supporting alternate distribution channels to get products to service delivery points, but these measures were described by interviewees as stopgap measures as opposed to sustainable systems improvements. Although IAP partners did provide technical assistance to individual countries and in regional workshops, capacity for national supply planning remains a barrier to achieving the forecast accuracy needed to ensure supply availability and maintain optimal national supply levels.

Overall, the most frequently cited challenge across country-level interviewees was supply shortages at both the national level and service delivery points. Despite some improvements in capacity for national supply planning, as well as in-country supply chain management, interviewees in the 3 case example countries noted a remaining need for overall system strengthening efforts to improve family planning supply chains and reduce shortages at service delivery points, as well as addressing global shortages that have resulted in stock-outs at central warehouses.

#### 4. Leveraged Existing Service Delivery Capacity

The IAP leveraged existing service delivery capacity investments in training, expanded delivery models, and conducted community awareness and sensitization activities from participating organizations. These investments, while valuable and important to the success of the IAP, were not wholly sufficient and gaps remain.

MOH stakeholders in each country coordinated with donors and implementing partners to align around national goals and implementation plans to scale up LARCs, including implants. These plans in most countries reflected broader family planning commitments and formed part of national costed implementation plans to achieve family planning goals. Thus, the IAP was able to align strategically to leverage existing in-country capacity and expertise to expand access to family planning.

**Leveraged Best Practices in Training.** Improved approaches were needed to achieve training goals with limited available resources, especially during the introduction of NXT and phaseout of Implanon classic. IAP partners tested various training models, identifying appropriate cost-effective solutions that combined on-the-job training with follow-up supportive supervision, mentoring, and coaching. These approaches were feasible as the updated product required minimal differences in insertion techniques and no substantial changes to patient counseling procedures.^16^ Interviewees estimated that these approaches reduced training costs by up to 60% when compared to traditional in-service training models. The approaches also improved providers’ competency to perform implant insertions and removals by allowing more opportunities to practice and maintain skills and establishing mechanisms for continued supportive supervision. The on-the-job training approach also minimized disruption of health service provision that often results from off-site training. IAP partners shared these best practices in training innovations through the Operations Group, enabling widespread dissemination across country partners in a timely manner.

Cost-effective solutions combined on-the-job training with follow-up supportive supervision, mentoring, and coaching.

Country stakeholders identified several key challenges related to provider training, including maintaining high-quality counseling and minimizing bias toward provision of specific methods, including implants. To overcome these challenges, ongoing embedded mentoring and coaching models were designed to reinforce training messages and improve quality counseling; however, these models do require ongoing resources to maintain their efficacy. Interviewees also identified a persistent system-wide challenge of retaining trained providers in contexts of high turnover of health personnel.

**Expanded Service Delivery Models.** Although some countries utilized outreach services and liberalized task sharing policies to expand access, other countries relied on their existing delivery capacity.

Outreach events and mobile clinics, serving high volumes of clients seeking family planning services, provided training opportunities for providers while also offering family planning access to women in remote areas where distance to clinics can pose a significant barrier.

The 2012 WHO task sharing recommendations^17^ identified opportunities for expanding the cadre of health workers that could provide implants and other family planning methods. These recommendations built from prior successful experiences in countries that had implemented task sharing policies as a means of increasing access to family planning. All 3 focus countries considered opportunities for task sharing of implants delivery, but barriers in Uganda and Kenya, particularly resistance from higher-level health care workers, have prevented policy change. Kenya allows insertion by nurses and midwives but has faced resistance when considering policy change to allow insertions by community health workers. Uganda has supported task sharing to allow nurses and midwives to perform insertions in policy documents, but they do not yet have legal protection to do so.^18^ In 2014, Nigeria successfully approved a task shifting policy allowing community health extension workers (CHEWs) to insert and remove implants. The current status of national scale-up efforts to train CHEWs in implants insertions and removals in Nigeria could not be confirmed for this evaluation. As one implementing partner in Nigeria explained:

*Changing the task shifting policy was a challenge, our nurses and midwives feel this was a duty for [they themselves]to provide so they were against it [task shifting to CHEWs] initially. However, we navigated through this by having an acceptable training manual for the entire health care system, which gave some level of comfort of any system we put in place. We also did a cost-benefit analysis and a big review of human resources for health and found that majority of health care workers were often CHEWs, so it prompted a conversation, that combined with data, was very successful.*

Implant availability in private sector for-profit facilities has not experienced the same growth seen in the public sector.^19^ Interviewees in the 3 case example countries noted this as a challenge to increasing access to implants. The price agreement applies to procurers purchasing for public sector delivery and delivery through SMOs who could charge a maximum fee for the product. Private for-profit providers cannot purchase implant commodities at the reduced price agreed to through the VGs. The higher price for these providers combined with competition with typically free or reduced-cost provision of implants in the public sector could undermine any financial incentive for these providers to offer implants. Without sustainable mechanisms for private for-profit providers to purchase at affordable prices, implants likely will not reach women through these channels. In Nigeria, nearly 60% of women access family planning through private medical sources,^20^ making access to the lower priced product for these providers an important factor to continue progress in increasing access to implants.

**Conducted Community Awareness and Sensitization Activities.** Demand generation was not an explicit objective of the IAP as there was an underlying assumption from the outset of latent demand for the method based on early experiences with introduction of free or highly subsidized implants.^3^ However, although country-level partners found that uptake was high as soon as providers were trained in some areas, they found low utilization and uptake in other areas. In general, partners found that the information and awareness gaps were around the benefits of family planning more broadly rather than implants specifically. Implementing partners were able to address these gaps through general information and communication efforts about family planning with an emphasis on the benefits of LARCs. These activities across the 3 case example countries used community health workers for knowledge and information sharing, as well as traditional and social media and mobile phone-based platforms. For example, successful activities were tailored to target audiences inclusive of male community members; religious, government and political leaders; health facility workers beyond service providers; and women in the community.

#### 5. Encouraged effective global and country-level collaboration and coordination

Coordination at the global and country level was an effective element of the IAP from the beginning. However, challenges arose in aligning around goals and commitments particularly as different partners were engaged at different levels and different phases over time. The IAP coordinated global stakeholders through a formal governance structure that facilitated information sharing and communication and drove joint problem solving. This agile, responsive governance structure was a critical factor in achieving implants scale-up goals. As a manufacturer representative explained:

*An aspect that made this unique and successful was the fact that the VG and the Oversight Board brought all the required experts around the table with 1 objective: scaling up access to this 1 method. People could act quickly and work together. Without that element, even with the VG, we would not have been as successful.*

The IAP used a formal, agile, and responsive governance structure that was critical to successfully achieving implants scale-up goals.

An example of how the governance structure supported problem solving was with the formation of the Implants Removals Task Force. Access to implants removal services was not a focus of initial IAP efforts, but over time, programmatic concerns about barriers to accessing removal services was escalated through the Operations Group. In response to this growing need, the IAP developed an Implants Removals Task Force in 2014 to coordinate efforts and provide programmatic guidance. This group identified standards that needed to be in place to assure quality removal services.^21^

As another example, working together with the 2 manufacturers and key procurers, the IAP Operations Group supported the development of standardized packaging, as opposed to customized packaging by procurer, for both implant products. This simplified packaging was a critical component for suppliers to meet the reduced price commitment, and at the same time, improved supply chain performance by allowing manufacturers to build up inventory without the need for customized packaging by procurer. Further, in 2015, several IAP partners, including UNFPA and CHAI, collaborated to develop a standardized consumables kit that combined the necessary supplies required for both implant insertion and removal.

According to interviewees, the collaboration and coordination among partners at the global level also contributed to coordination among partners at the country level. Coordination efforts led by government stakeholders that brought together the MOH, donors, implementing partners, SMOs, and the private sector around national scale-up plans were identified as a key success factor across case example countries. This coordination was important at both the national and subnational levels and served to strengthen efforts and minimize duplication across family planning programs.

However, the partnership also faced key challenges in terms of accountability and commitments toward common goals, particularly during the early phase of the partnership. The VG agreement negotiation relied on a small group of stakeholders to align on key parameters to ensure confidentiality and minimize potential conflict of interest. The broader set of partners were engaged at different levels and at different phases of the negotiation process to secure commitments and achieve alignment. The VG agreements inherently created the potential for tensions between those accountable for the guarantee and those providing the majority of the procurement resources. This tension, which was noted by several interviewees, was addressed to some extent through the governance mechanisms but remained a challenge throughout the life of the partnership.

### Sustainability

#### 6. Continued Efforts and Expanded Resources

The outlook for sustainability of the gains realized by the IAP is strong given commitments made by partners and the plans to continue and expand resources. However, price remains a concern among many key stakeholders. An internal sustainability assessment of the IAP completed in 2017 found that support for supply planning, operations, and market dynamics at the global level should continue to sustain progress, but that these functions would need to be integrated into existing institutions given the formal end of the IAP in 2018. To support such a transition, these functions will need to be expanded to include family planning methods generally, not just implants.

Several mechanisms from the IAP have already evolved into institutionalized systems. CSP, as a workstream of the RHSC, continues, and efforts are underway to transition the data visibility tools and processes to support supply coordination to the Global Family Planning Visibility and Analytics Network. This platform will capture and use supply chain data from multiple sources and organizations to provide enhanced visibility for decision making across multiple family planning products. Discussions are also ongoing with the United States Agency for International Development (USAID) regarding the feasibility of folding the IAP’s Operations Group into USAID’s Method Choice community of practice (formerly LARC community of practice). The Implants Removal Task Force is also in the process of evolving its mandate to include IUDs.

At a country level, multiple factors point to the likelihood of maintaining efforts initiated under the IAP to increase access to implants. Countries have developed and adapted national implants training methods and curricula for health workers, and in the case of Uganda, included implants training into a standard curriculum for health worker education programs.^22^ In addition, the family planning dashboards that support linking of trained providers with family planning commodities are in use at a national level in several FP2020 countries, significantly improving forecasting efforts. Finally, coordination between in-country implementing partners and with the MOH has improved, which is essential to manage resources and organize programmatic efforts.

The sustained low unit price of the implants products was consistently cited by interviewees as the most critical factor to maintaining achievements at the country level. Although the VG ended in 2018, manufacturers have committed to maintaining the current price through 2023. While this can be considered a key success of the IAP, the price reduction needs to be sustained beyond this period to ensure long-term implants access, and manufacturers will ultimately need an effective business case to do so. An equally critical factor to ensuring sustainability is maintaining government commitment and political will to continue efforts to improve access to implants, and the family planning method mix more broadly. Key remaining challenges that were identified to maintain and expand progress at the country level are to ensure capacity and access to affordable commodities for the private sector and to ensure training and human resource considerations to meet the growing demand for implants removals.

A key success of the IAP was manufacturer’s commitment to maintain the current implant price until 2023.

### Summary

Overall, this evaluation demonstrates that the IAP was relevant to the needs of the family planning community and effective at achieving its objectives. However, challenges do remain. Evidence also suggests that progress will be sustained over time, with continued global and country efforts. Focused monitoring will be necessary to maintain progress, particularly to ensure long-term affordability and availability of the product in the global market.

## DISCUSSION

The findings above identify key lessons in terms of how success was achieved at the global and country level and provide valuable insights to inform recommendations for global and country stakeholders in the broader family planning field. In considering how to apply the lessons learned from the IAP, it is also important to consider the context in which the IAP operated and recent global health trends that could impact the relevance, effectiveness, and sustainability of similar efforts in the future. The VG was a unique solution to address a unique problem of unmet demand due to the high price of implants. The price solution was complemented by other investments to address access barriers and enabled by the broader context in which the IAP operated, including the growing momentum in the family planning field. Emerging trends that will be important to consider for future efforts include positioning family planning as part of Universal Health Coverage, increasing focus on self-care interventions for sexual and reproductive health, shifting funding toward domestic and pooled financing sources, and creating a new product pipeline that could result in potential product introduction fatigue.

### Recommendations

1. Integrate and align method-specific efforts in support of broader family planning goals to achieve sustainable success and drive progress at both the global and country level. IAP efforts to leverage and integrate with the broader family planning global architecture were critical to success both in increasing access to implants and improving the broader enabling environment for contraceptives. The integration with the global family planning ecosystem took time to evolve from initial perceptions of the IAP being a method-specific effort. The partnership made concerted efforts to become more inclusive and align with the overall global family planning agenda. At the country level, interviewees noted that positioning this effort within ongoing family planning and LARC scale-up initiatives was critical from the outset, allowing partners to leverage existing political will toward increasing access to LARCs. This alignment enabled the IAP to both achieve its goals and contribute to the broader family planning goals to reach additional users. This integration is also critical to sustaining progress after the program ends.

2. Support and align method-specific efforts with country family planning policy and implementation plans led by government, informed by evidence and starting from a rights-based approach. Efforts to scale up contraceptive products should be embedded within existing national efforts, creating country ownership and building political will with respective MOHs and local service delivery partners. For the IAP, it was essential that procurers, technical assistance providers, and implementing partners collaborated effectively with their government counterparts, engaged in regular meetings and ongoing dialogue, and supported the development of national policies and guidelines. In-country implementers can also use evidence from successful smaller-scale introduction efforts to advocate for national policy change. For example, in Nigeria, in-country implementing partners were able to build on evidence from successful pilot projects to support the national government to develop a task sharing policy allowing community health workers to insert implants. Ultimately, efforts to scale up access to implants must be part of a well-balanced contraceptive method mix, ensuring a rights-based approach to increasing women’s access to family planning.

3. For price reductions to truly increase access to commodities, effective partnerships and complementary investments are needed. Current trends in family planning financing include increasing reliance on domestic resources and shifts in donor resources toward pooled financing mechanisms, such as the Global Financing Facility.^23^ These trends will make efforts to reduce prices and increase access to low-cost contraceptives even more critical. Ultimately, VGs are only 1 option to reduce price and increase access to family planning commodities that must be considered carefully in the unique context of that specific commodity, along with other options such as pooled procurement mechanisms, direct buy-down of price, or direct investment in suppliers.^4^^,^^9^

In the case of the IAP, implant manufacturers viewed their engagement in the VG as a success, both in building a partnership that was successful in reaching its goals and in providing a great example of corporate social responsibility efforts. However, a VG should only be employed with careful considerations for resource requirements and with a clear understanding of the value from both a business and social perspective. In the case of implants, the lower price allowed existing resources to be stretched further. At the same time, a price reduction alone is unlikely to be sufficient to drive significant scale-up. The complementary activities to increase access and improve the enabling environment at the country level were critical to success for the IAP, but represented a substantial investment of time and resources. For example, the price reduction for implants changed the engagement of manufacturers and shifted much of the responsibility and associated resources for training and product introduction to donors and governments.

4. Engage partners early and with a high degree of transparency to ensure alignment around commitments and accountability to common goals for successful multistakeholder partnerships. Approaches that engage and coordinate partners across sectors and stakeholder groups are increasingly relevant to the family planning field and can drive progress through leveraging existing resources. The collaborative efforts of IAP partners allowed each partner to contribute resources, knowledge, and skills in a coordinated approach and with a dedicated forum for problem solving that enabled greater impact than if partners had contributed the same resources separately. Early engagement of all relevant stakeholders is key to ensure alignment around common goals, resource requirements, and individual partner commitments to achieve goals. This engagement is particularly critical with approaches that have an inherent risk of tension given the multipartner accountability toward VGs. Formalized mechanisms that support transparency, data sharing, and communications across stakeholders can drive joint problem solving and increase coordination in support of common goals. This recommendation aligns with findings in published literature on the core components required for collective impact, including a common agenda and shared measurement systems, both critical elements for the IAP’s success.^24^

5. Increase data visibility across all levels of the supply chain to better match supply with demand and improve forecasting abilities to smooth overall procurement. At both the global and country levels, increasing data visibility was critical to reducing supply chain disruptions. At the global level, this effort included sharing order data between individual manufacturers and procurers to better coordinate orders and meet country needs, while ensuring this data was not shared between manufacturers to maintain confidentiality agreements. Going forward, the Global Family Planning Visibility and Analytics Network will continue to play an important role in data visibility efforts. At the country level, increasing supply data visibility and providing managers with access to these data combined with training and consumption data was a successful strategy to improve supply availability at service delivery points. However, the systems-level constraints around national and local commodity distribution systems are significant and represented a barrier to achieving the IAP objectives across case example countries. Beyond data visibility, investments and opportunities to improve overall supply chain systems should be considered as a part of any effort to scale new and underutilized family planning products, given the challenges that were faced for the IAP at the country level.

6. Design training programs with scale-up and sustainability in mind. Innovative training approaches were critical to the success of the IAP by reducing associated costs and allowing limited resources to stretch further. Successful training approaches also incorporated ongoing support for providers to maintain skills for both insertions and removals and embedded training capacity within facilities to support and mentor new staff. Integrating new content within nurse and midwife curricula goes even further to develop and sustain provider capacity over the long term.

7. Conduct sensitization and awareness raising for family planning and the full range of methods as a key component to ensuring women and couples can exercise free and informed contraceptive method choice. Efforts to increase scale-up and access to any modern contraceptive method must be grounded in the reality that women and couples across the globe lack access to necessary family planning information and often face significant barriers in accessing high-quality family planning care.^25^ Global IAP efforts correctly focused on latent demand for implants, and the lower price allowed the family planning community to procure the quantities needed to meet that demand. However, country partners identified an ongoing need for client, provider, and community sensitization and awareness activities targeted at the benefits of family planning use and how to access all contraceptive methods and not just focused on increasing use of any specific method. These efforts are critical, not only to ensure women have knowledge of family planning methods, but also to enable women to access contraceptives within the context of free and informed choice.

### Limitations

This evaluation faced limitations that should be considered in interpreting the findings. First, the country stakeholder interviewees were limited to only 3 countries. The evaluation wanted to capture a variety of perspectives within a given country, but many other countries increased implants uptake during this period. Additionally, scheduling conflicts beyond our control precluded MOH officials in 2 case example countries from participation in interviews, which could have provided valuable perspectives to the evaluation. However, the evaluation team spoke to multiple implementing partners and donors in both countries who had worked closely with the MOH during the time period of the IAP. Second, the evaluation was conducted by Global Impact Advisors, who had previously served as the Secretariat for the IAP. However, the evaluation team was led by 2 individuals who had not previously played any role in the IAP, thus reducing the likelihood of any bias in interpreting evaluation findings.

## CONCLUSIONS

The IAP was one of the largest global efforts to reduce the price of and increase access to implants by building a public- and private-sector collaboration that focused on systems change in the family planning field. Over 6 years, the IAP supported tremendous progress in increasing access to implants for women in the world’s poorest countries. As an outcome of this partnership effort, tools, systems, and capacity were built that can be leveraged to facilitate future introductions of new and underutilized contraceptive products. These include family planning dashboards that support alignment of commodities with trained providers, innovative and cost-effective training approaches, and mechanisms to support coordination and data sharing among procurers, donors, manufacturers and implementing partners.

To understand the full extent of its impact, the IAP must be placed within the overall context of the market for contraceptives and the various influencing factors that may have shaped that market. However, the relatively short time frame of the IAP makes it difficult to draw conclusions about the evolution and long-term sustainability of the market. Thus, future research should endeavor to understand the complexity of the market for implants and other contraceptives, the drivers of change over time, and the factors that are likely to influence the sustainability of prices and supply after current agreements end in 2023.

Program designers and implementers across the family planning field can use the lessons learned from the IAP to improve collaboration, build new and strengthen existing supply chain and service delivery efforts, and support effective public-private collaborations to introduce and scale up new and underutilized contraceptive methods.

## Notes

**Peer Reviewed**

**First published online:** May 28, 2020

## References

[1] Family Planning’s Return on Investment. FP2020 website. Accessed July 16, 2019. https://www.familyplanning2020.org/sites/default/files/Data-Hub/ROI/FP2020_ROI_OnePager_FINAL.pdf

[2] Ngo TD, Nuccio O, Reiss K, Pereira SK. *Expanding Long-Acting and Permanent Contraceptive Use in Sub-Saharan Africa to Meet FP2020 Goals*. https://www.mariestopes.org/resources/expanding-long-acting-and-permanent-contraceptive-use-in-sub-saharan-africa-to-meet-fp2020-goals/. Published 2013. Accessed July 16, 2019.

[3] Duvall S, Thurston S, Weinberger M, Nuccio O, Fuchs-Montgomery N. Scaling up delivery of contraceptive implants in sub-Saharan Africa: operational experiences of Marie Stopes International. Glob Heal Sci Pract. 2014;2(1):72–92. 10.9745/ghsp-d-13-00116. 25276564 PMC4168608

[4] United Nations Commission on Life-Saving Commodities for Women and Children. *Commissioner’s Report September 2012*. https://www.unicef.org/media/files/UN_Commission_Report_September_2012_Final.pdf. Published 2012. Accessed July 16, 2019.

[5] Jacobstein R. Liftoff: The blossoming of contraceptive implant use in Africa. Glob Heal Sci Pract. 2018;6(1):17–39. 10.9745/GHSP-D-17-00396. 29559495 PMC5878070

[6] Bank D. Guaranteed Impact. https://ssir.org/articles/entry/guaranteed_impact. Published 2016. Accessed June 24, 2019.

[7] Implants Access Program: Expanding Family Planning Options for Women. FP2020. http://ec2-54-210-230-186.compute-1.amazonaws.com/wp-content/uploads/2016/03/IAP_two_pager_2016-REV-jan-21.pdf. Published January 2016. Accessed June 24, 2019.

[8] Bank D. Guaranteed impact. https://ssir.org/articles/entry/guaranteed_impact. Published 2016. Accessed June 24, 2019.

[9] United Nations Population Fund (UNFPA) Procurement Services. Reproductive Health Interchange. Accessed July 26, 2019. https://www.unfpaprocurement.org/rhi-home

[10] Akhlaghi L, Heaton A, Chandani Y. Are procured quantities of implants adequate and appropriate? Modeling procurement, inventory, and consumption of contraceptive implants during rapid uptake. Glob Heal Sci Pract. 2019;7(2):240–257. 10.9745/GHSP-D-19-00017. 31249021 PMC6641805

[11] Implants Access Program: Expanding family planning options for women. FP2020 website. http://www.familyplanning2020.org/sites/default/files/Our-Work/ppfp/2018%20IAP%202%20pager_VF.pdf. Published November 2018. Accessed June 24, 2019.

[12] *Contraceptive Implants Product Brief*. https://www.rhsupplies.org/uploads/tx_rhscpublications/RHSC_implants_br.pdf. Caucus on New and Underused Reproductive Health Technologies. Reproductive Health Supplies Coalition. Published January 2011. Accessed July 24, 2019.

[13] Commitment makers. FP2020. http://www.familyplanning2020.org/countries. Published 2018. Accessed July 24, 2019.

[14] *Contraceptive Implants Product Brief*. Reproductive Health Supplies Coalition. https://www.fhi360.org/sites/default/files/media/documents/rhsc-brief-contraceptive-implants_A4.pdf. Published 2013. Accessed July 24, 2019.

[15] Darroch J, Singh S. *Estimating Unintended Pregnancies Averted from Couple-Years of Protection (CYP)*. https://www.guttmacher.org/sites/default/files/page_files/guttmacher-cyp-memo.pdf. Published 2011. Accessed September 12, 2019.

[16] Jhpiego. *Providing Contraceptive Implants Learning Resources Package*. Baltimore, MD: Jhpiego; 2015. http://reprolineplus.org/resources/implants-LRP

[17] World Health Organization (WHO). *Optimizing Health Worker Roles to Improve Access to Key Maternal and Newborn Health Interventions Through Task Shifting*. https://www.who.int/reproductivehealth/publications/maternal_perinatal_health/978924504843/en/. Published 2012. Accessed June 24, 2019. 23844452

[18] Moses M, Nassimbwa J, Sekimpi C, Kyateeka FN. Exploring the regulation of task sharing for access to family planning services in Uganda. Lupine J Nursing Health Care. 2018;1(3). 10.32474/LOJNHC.2018.01.000112

[19] Riley C, Garfinkel D, Thanel K, et al. Getting to FP2020: Harnessing the private sector to increase modern contraceptive access and choice in Ethiopia, Nigeria, and DRC. PLoS One. 2018;13(2):e0192522. 10.1371/journal.pone.0192522. 29444140 PMC5812628

[20] Federal Republic of Nigeria, National Population Commission; ICF International. *Nigeria Demographic and Health Survey 2013*. http://dhsprogram.com/pubs/pdf/FR293/FR293.pdf. Published 2014. Accessed July 14, 2019.

[21] Christofield M, Lacoste M. Accessible contraceptive implant removal services: an essential element of quality service delivery and scale-up. Glob Heal Sci Pract. 2016;4(3):366–372. 10.9745/GHSP-D-16-00096. 27577239 PMC5042693

[22] Mugore S, Mwanja M, Mmari V, Kalula A. Adaptation of the training resource package to strengthen preservice family planning training for nurses and midwives in Tanzania and Uganda. Glob Heal Sci Pract. 2018;6(3):584–593. 10.9745/GHSP-D-18-00030. 30166327 PMC6172108

[23] FP2020: Catalyzing collaboration 2017–2018. FP2020. http://2017-2018progress.familyplanning2020.org/content/finance#anchor-sub_chapters-370. Published 2018. Accessed July 24, 2019.

[24] Kania J, Kramer M. Collective impact. https://ssir.org/articles/entry/collective_impact. Published 2011. Accessed July 26, 2019.

[25] UNFPA Division of Communication and Strategic Partnerships. State of world population 2019. https://www.unfpa.org/swop-2019. Published 2019. Accessed July 26, 2019.

